# Pelvicalyceal Squamous Cell Carcinoma: Incidental Diagnosis With Liver Metastasis on Follow Up

**DOI:** 10.7759/cureus.18345

**Published:** 2021-09-28

**Authors:** Zini Chaurasia, Swapnil Agarwal, Renu Gupta, P.S. Khatana

**Affiliations:** 1 Pathology, Dr. Baba Saheb Ambedkar (BSA) Medical College and Hospital, New Delhi, IND; 2 Urology, Dr. Baba Saheb Ambedkar (BSA) Medical College and Hospital, New Delhi, IND

**Keywords:** nephrolithiasis, metastasis, incidental, scc, renal pelvis

## Abstract

Primary squamous cell carcinoma (SCC) of the renal pelvis is a rare malignancy. We report a case of a 43-year-old male presenting with stones and a non-functioning kidney. Incidental SCC was diagnosed histopathologically on nephrectomy. The patient then returned with metastasis to the liver after one year. Nephrolithiasis is the most important risk factor implicated in renal SCC; hence, awareness of this rare entity in a patient of long-standing nephrolithiasis is important.

## Introduction

Squamous cell carcinoma (SCC) denotes cancer where tumor cells resemble stratified squamous epithelium, which commonly occurs in the oral cavity, cervix, skin, lung, etc. [1]. Primary SCC of the renal pelvis is rare with incidence reported to be about 0.8% [2]. Nephrolithiasis with superadded chronic infection is the most important predisposing factor. It usually manifests at advanced stages due to its vague clinical presentation and non-specific radiological findings. Here, we report a case of renal pelvic SCC incidentally diagnosed on histopathology in a 43-year-old male who underwent nephrectomy for a non-functioning kidney due to nephrolithiasis. After nephrectomy, the patient again presented with retroperitoneal mass and liver metastasis on follow-up.

## Case presentation

A 43-year-old male patient came to surgery OPD with complaints of dull and intermittent right side flank pain for several months. The patient also complained of decreased urine output. The patient did not give any other significant history. The X-ray and ultrasound of the abdomen revealed multiple stones in the right kidney and hydronephrosis. Blood urea and serum creatinine were also raised. A clinical diagnosis of non-functioning right kidney secondary to nephrolithiasis was made and a nephrectomy was performed.

**Figure 1:**
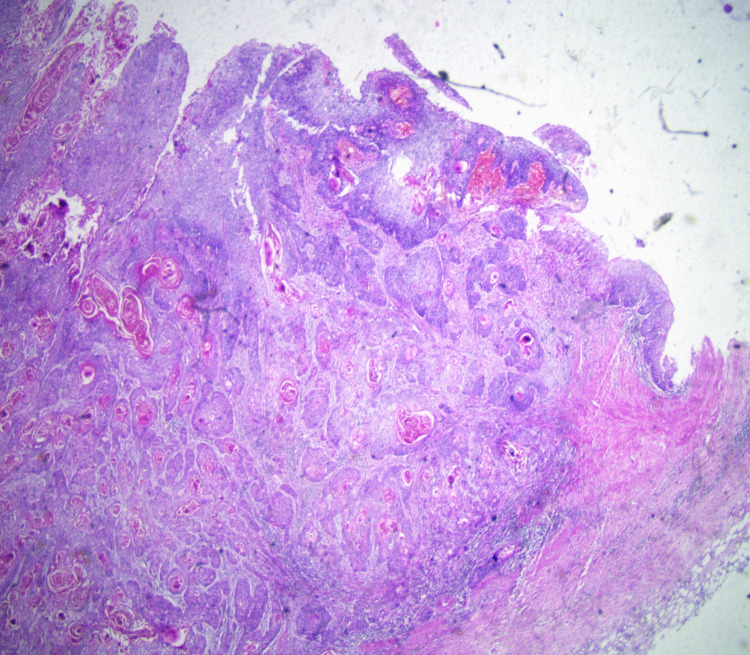
H&E, 4× metaplastic epithelium with transformation to squamous cell carcinoma, renal pelvis. H&E: hematoxylin and eosin.

A total of 53 nephrectomy specimens were received in our department in the last four years for chronic kidney disease, of which only one showed renal pelvic SCC. A nephrectomy specimen in 10% formalin was received in the Department of Pathology, Dr. Baba Saheb Ambedkar Hospital with an attached ureter measuring 10 × 6 × 3 cm^3^. The outer surface was nodular covered with adherent perinephric fat. On serial slicing, it was observed that the kidney had converted into a multilocular cystic structure with thinned out cortex and cortico-medullary junction could not be appreciated. A solid, greyish white growth in the renal pelvis measuring 3 × 2.5 cm^2^ was also seen which was infiltrating the perirenal adipose tissue. Multiple stones were seen in the pelvis and in the dilated calyceal spaces. Sections from the growth revealed histomorphology consistent with well-differentiated SCC with areas of necrosis and hemorrhage (Figure 1).

**Figure 2:**
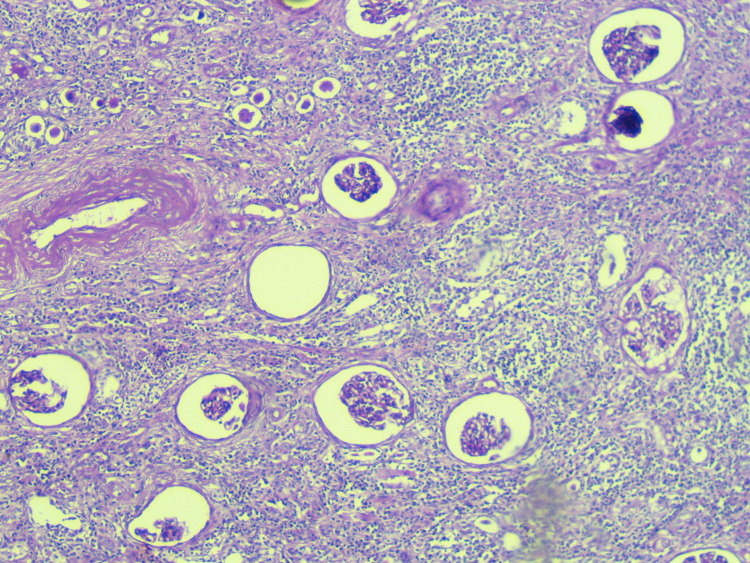
H&E, 40× - Atrophy of tubules with thyroidisation, chronic interstitial inflammatory infiltrate, thickened blood vessels suggestive of chronic pyelonephritis. H&E: hematoxylin and eosin.

The tumor was seen infiltrating the perinephric fat. Sections from adjacent renal parenchyma showed features of chronic pyelonephritis (Figure 2).

**Figure 3:**
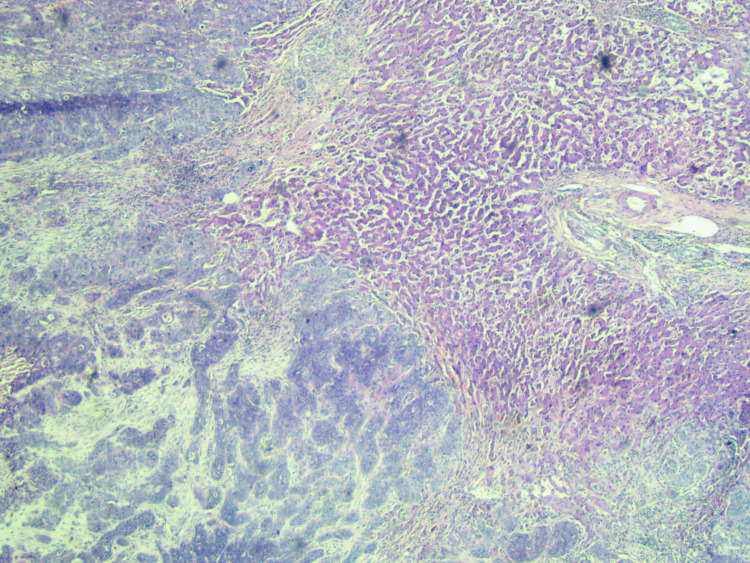
H&E, 10× - Metastasis in the liver. H&E: hematoxylin and eosin.

The ureteric end was free of tumor, however, the vessels could not be identified due to the location of the tumor. Taking note of all the findings, a final diagnosis of SCC, renal pelvis, pT3NxMx, and right kidney was made. The patient returned to the hospital one year after, with complaints of vomiting, weight loss, and right side abdominal pain. CT scan of the abdomen revealed a mass involving the right retroperitoneal area extending up to the right lobe of the liver. The whole mass along with the right lobe of the liver (partial) was resected and sent for histopathological examination. A globular soft tissue mass with a skin flap was received measuring 15 × 14 × 10 cm^3^. The mass measured 12 × 11 × 9 cm^3^. It was 1 cm away from the skin. Resected liver sent separately showed a growth measuring 5 × 3 × 2 cm^3^. On the cut section, the tumor had a variegated appearance with necrotic and hemorrhagic areas. Multiple sections from the tumor showed features of moderately differentiated SCC (Figure 3).

The overlying skin was free from any tumor. Sections from the liver showed infiltration by a similar tumor. The patient was lost to follow up after discharge from the hospital.

## Discussion

Transitional cell carcinoma is the most common type of carcinoma arising from the renal pelvis [3]. SCC is extremely rare in the renal pelvis accounting for less the 0.8% of renal tumors [4]. Several authors have reported renal pelvic SCC with nephrolithiasis. A comparison between various similar case reports and present case report is shown in Table 1. Various other risk factors have been associated with renal pelvis SCC apart from nephrolithiasis. Chronic pyelonephritis, vitamin A deficiency, smoking, and several endogenous/exogenous chemicals being a few of them [4]. It has been postulated that the presence of long-standing nephrolithiasis initiates squamous metaplasia of urothelium due to chronic irritation which further develops into SCC [5]. This patient also had a long-standing history of the presence of stones which ultimately led to a nonfunctioning kidney for which nephrectomy was performed. In our patient, the diagnosis of SCC was made only on histopathology as radiological examination revealed only multiple stones, hydronephrosis, and non-functioning kidney. Non-specific radiological findings in renal SCC have been reported by various other authors [6]. Findings of common renal diseases like hydronephrosis, stones, pyelonephritis, etc., became the indication for this surgery. Kartal et al. have described in their case report that their patient after nephrectomy and on cisplatin therapy subsequently succumbed after 17months [7]. Kalayci et al. mentioned in their article that the aggressive nature of renal pelvis SCC combined with non-specific radiological features leads to the patient being diagnosed at stage pT3 or advanced stages and thus the survival time decreases significantly for these patients [8]. In our case also, the patient was diagnosed at pT3 and came back with recurrence after one year with retroperitoneal recurrence and metastasis to the liver. The reason for such a large metastatic mass presenting in our case is because of the fact that the patient was non-compliant during follow-up. Diagnosis of SCC remains unsuspected and is sometimes diagnosed only post-surgically as an incidental finding inpatient operated for other renal diseases [9,10]. To the best of our knowledge, only Xiao et al. and Jongyotha and Sriphrapradang have reported one case report of recurrence in the liver similar to our case [11,12]. The low reporting of recurrence may be because the patient rarely survives because of the poor prognosis of renal SCC. In the studies that we compared follow-up was there for six months, however, Jongyotha and Sriphrapradang have mentioned an average survival time of seven months for renal pelvis SCC after diagnosis [12]. Though our patient survived for a year the recurrence caused significant morbidity and reduced his quality of life.

**Table 1:** Review of literature: pelvicalyceal squamous cell carcinoma.

Author [Reference]	Year	Age	Initial presentation	Location	Invasion/metastasis	Treatment	Recurrence/follow up
Sun and Li [5]	2020	66	Low back pain	Right	Skin metastasis	Open nephrectomy	Died 3 months postoperatively
Kartal et al. [7]	2018	38	Right side flank pain	Right	No	Radical nephrectomy	Died post- operatively 17 months
Paonessa et al. [9]	2010	70	Abdominal pain	Left	NA	Radical nephrectomy	No follow up
Ogawa et al. [10]	2014	71	Lower back pain and hematuria	Left	NA	Total nephrourectomy	6 months no recurrence
Xiao et al. [11]	2015	55	Dull lumbar region pain	Right	Yes, liver metastasis	Radical nephrectomy	Died 1 year later after liver metastasis
Xiao et al. [11]	2015	61	Bilateral flank pain	Right	No	Radical nephrectomy	Died during chemotherapy
Jongyotha and Sriphrapradang [12]	2015	79	Abdominal pain, vomiting, anorexia	Right	Yes, liver metastasis	Radical nephrectomy	Passed away one month later.
Present report	2021	43	Flank pain	Right	Liver metastasis after 1 year	Simple nephrectomy, excision of the mass with partial resection of right liver lobe	Recurrence after 1 year

## Conclusions

Renal pelvis SCC because of its aggressive nature and unfavorable prognosis needs to be suspected earlier. The clinicians need to investigate older patients of nephrolithiasis vigorously as it is common and also the most important factor implicated in renal pelvis SCC. Early detection and removal of stones will remove the inciting factor for SCC. Hence, awareness about the involvement of this rare site is necessary. Early suspicion for SCC, diagnosis and treatment can prolong life and improve survival chances for patients.
